# Chronic antidepressant potentiates spontaneous activity of dorsal raphe serotonergic neurons by decreasing GABA_B_ receptor-mediated inhibition of L-type calcium channels

**DOI:** 10.1038/s41598-017-13599-3

**Published:** 2017-10-19

**Authors:** Nozomi Asaoka, Naoya Nishitani, Haruko Kinoshita, Hiroyuki Kawai, Norihiro Shibui, Kazuki Nagayasu, Hisashi Shirakawa, Takayuki Nakagawa, Shuji Kaneko

**Affiliations:** 1 Department of Molecular Pharmacology, Graduate School of Pharmaceutical Sciences, Kyoto University, 46-29 Yoshida-Shimoadachi-cho, Sakyo-ku, Kyoto, 606-8501 Japan; 2 Department of Clinical Pharmacology and Therapeutics, Kyoto University Hospital, 54 Shogoin-Kawahara-cho, Sakyo-ku, Kyoto, 606-8507 Japan

## Abstract

Spontaneous activity of serotonergic neurons of the dorsal raphe nucleus (DRN) regulates mood and motivational state. Potentiation of serotonergic function is one of the therapeutic strategies for treatment of various psychiatric disorders, such as major depression, panic disorder and obsessive-compulsive disorder. However, the control mechanisms of the serotonergic firing activity are still unknown. In this study, we examined the control mechanisms for serotonergic spontaneous activity and effects of chronic antidepressant administration on these mechanisms by using modified *ex vivo* electrophysiological recording methods. Serotonergic neurons remained firing even in the absence of glutamatergic and GABAergic ionotropic inputs, while blockade of L-type voltage dependent Ca^2+^ channels (VDCCs) in serotonergic neurons decreased spontaneous firing activity. L-type VDCCs in serotonergic neurons received gamma-aminobutyric acid B (GABA_B_) receptor-mediated inhibition, which maintained serotonergic slow spontaneous firing activity. Chronic administration of an antidepressant, citalopram, disinhibited the serotonergic spontaneous firing activity by weakening the GABA_B_ receptor-mediated inhibition of L-type VDCCs in serotonergic neurons. Our results provide a new mechanism underlying the spontaneous serotonergic activity and new insights into the mechanism of action of antidepressants.

## Introduction

The serotonergic system plays an important role in regulating a wide variety of brain functions, such as mood and cognition^1^. Among the serotonergic nuclei, the DRN regulates mood- and emotion-related behaviors, and the functional changes in this area are associated with various mental illnesses. DRN serotonergic neurons have slow and regular firing activity when recorded *in vivo* ^2^, suggesting that this tonic firing plays important roles for maintaining mood. Supporting this hypothesis, a growing body of evidence implicates that a change in the activity of DRN serotonergic neurons alters affection status^3–5^, while the mechanisms for modulating serotonergic activity are not fully uncovered.

Despite the fact that serotonergic neurons are tonically active when recorded *in vivo*, most of the previous *ex vivo* electrophysiological analyses used pharmacological and/or electrical stimulations to generate continuous firing because of the difficulty in maintaining the spontaneous activity of serotonergic neurons in acute brain slices^2,6,7^. In this context, it was widely believed that the excitatory inputs from another brain area, such as the prefrontal cortex and locus coeruleus, are necessary for the tonic firing activity of serotonergic neurons, while previous study suggests the existence of intrinsic pacemaker mechanisms in serotonergic neurons^8^. Until now, this contradiction between *in vivo* and *ex vivo* studies was not resolved.

Like most neurons, serotonergic neurons receive local GABAergic inhibitory inputs^9^. Recently, we investigated the local feedback circuit between DRN serotonergic neurons and GABAergic interneurons and found that continuous GABAergic inhibition maintains serotonergic activity^10^. GABA_A_ receptor-mediated ionotropic inputs are well-studied, while several lines of evidence suggest that postsynaptic GABA_B_ receptors may contribute to the modulation of serotonergic neurons^4,11^. Furthermore, chronic stress increases GABAergic neuronal activity and GABA_B_ receptor expression in the DRN^12,13^. These observations indicate the possibility that GABA_B_ receptor-mediated signaling contributes to the modulation of the baseline activity of serotonergic neurons, while little is known about its molecular mechanisms of GABA_B_ receptor-mediated inhibition of serotonergic neurons.

Most of the clinically-used drugs for psychiatric disorders such as selective serotonin reuptake inhibitors (SSRIs) modulate the serotonergic function of the brain^14^, while the precise mechanisms of such serotonergic drugs remain to be elucidated. Antidepressants have a delayed onset of action, suggesting that chronic antidepressant treatment-induced cellular and synaptic changes are necessary for the therapeutic action^15,16^. Consistent with these reports, we previously showed that chronic treatment with antidepressants enhances serotonin release *in vitro* ^17,18^. These findings suggest the possibility that chronic treatment with antidepressant potentiates serotonergic activity.

In the present study, by using modified *ex vivo* electrophysiological recording method, we could record serotonergic spontaneous firing activity even without any stimulations. This spontaneous firing activity was mainly regulated by L-type voltage-dependent Ca^2+^ current, which was continuously inhibited by GABA_B_ receptor-mediated signaling. Moreover, chronic administration of an antidepressant disinhibited the serotonergic spontaneous firing activity by weakening the GABA_B_ receptor-mediated continuous inhibition. These results offer a new mechanism for the GABAergic inhibition of DRN serotonergic neurons, which was responsive to chronic antidepressant treatment.

## Results

### DRN serotonergic neurons spontaneously generate action potentials in *ex vivo* electrophysiological recordings

**Figure 1:**
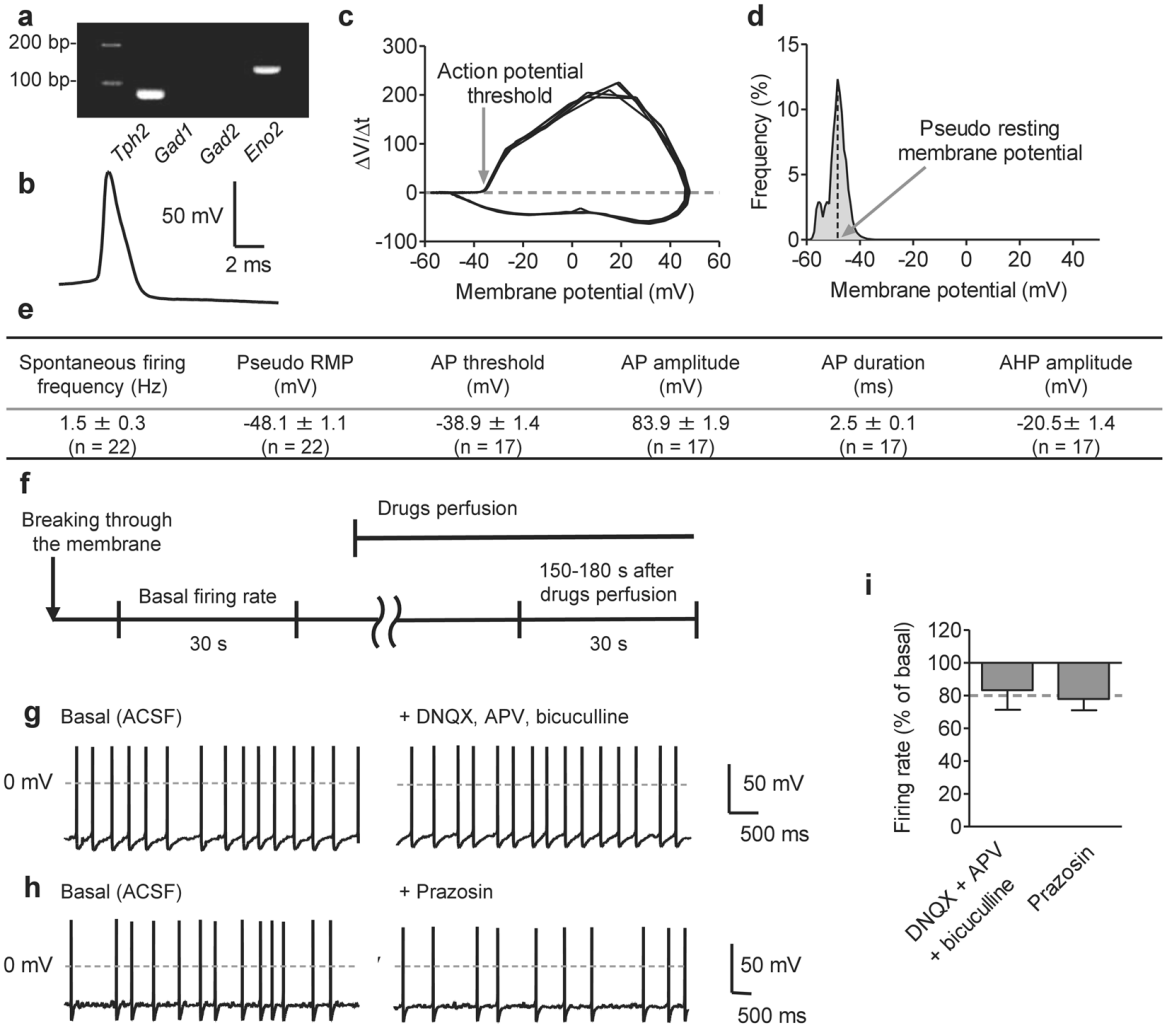
Serotonergic neurons in the dorsal raphe nucleus (DRN) show spontaneous firing activity. (**a**) Representative cropped image of single-cell reverse transcription polymerase chain reaction after whole-cell recording. Tryptophan hydroxylase 2 (*Tph2*) mRNA was used as a marker of serotonergic neurons. Glutamate decarboxylase 1 and 2 mRNA (*Gad1* and *Gad2*), markers of GABAergic neurons, were used as negative controls. Gamma-enolase mRNA (*Eno2*), a marker for neurons, was used as a positive control. Uncropped image was shown in Supplementary Fig. S1. (**b**) Representative trace of the action potential (AP) recorded from DRN serotonergic neurons. Serotonergic neurons showed a wide action potential and a long-lasting after hyperpolarization. (**c**) Representative phase plane plot of membrane potential *vs.* its derivative with respect to time (dV/dt). Five APs from one neuron was plotted. (**d**) Representative membrane voltage histogram. The higher voltage peak was considered as pseudo resting membrane potential (RMP). (**e**) Electrophysiological characters of 22 serotonergic neurons from 7 mice. Recordings were performed in normal ACSF condition without any drug or electrical stimulation. AHP; afterhyperpolarization. (**f**) Time course of recording the effects of drug perfusion. Spontaneous firing was recorded for 30 s before and after drug application, and changes in the firing rate were calculated. (**g,h**) Representative traces of the spontaneous firing before (left) and after (right) the application of DNQX (20 μM), APV (50 μM) and bicuculline (20 μM) (**g**) or prazosin (1 μM) (**h**). (**i**) The changes in the spontaneous firing rate before and after the application of DNQX (20 μM), APV (50 μM), and bicuculline (20 μM), or prazosin (1 μM). (DNQX + APV + bicuculline, *n* = 4 neurons from 3 mice, *P* = 0.2545 by paired *t*-test; prazosin, *n* = 3 neurons from 2 mice, *P* = 0.0855 by paired *t*-test). Data are presented as the mean ± S.E.M.

To examine control mechanisms for DRN serotonergic activity, we modified the recording method, which enables recording serotonergic spontaneous firing activity. While most of previous researches pointed out that serotonergic neurons are silent in *ex vivo* recordings^2,6^, recent study suggests that part of serotonergic neurons (~50%) showed spontaneous firing activity in “high quality” brain slices^7^. To increase spontaneously active serotonergic neurons, we prepared coronal brain slices with strictly controlled knife speed and vibration (see Methods) to avoid pressure-induced neuronal damage. Additionally, we used NMDG-based cutting solution, which are suitable for slicing adult brains^19^. By these modifications, we achieved recording spontaneous firing activity from more than 75% of DRN serotonergic neurons, which expressed *Tph2* mRNA (Fig. 1a,e; Supplementary Fig. S1). Similar to previous reports^2^, serotonergic neurons showed wide action potential (AP) and large afterhyperpolarization (AHP) amplitude (Fig. 1b,e). To examine AP threshold and resting membrane potential (RMP) in spontaneously active serotonergic neurons, we used phase plane plot and voltage histogram, respectively^20^ (Fig. 1c,d). In our methods, most of firing characters were essentially similar to those reported previously^7,21^, while slight depolarization of RMP (−48.1 ± 1.1 mV) and low AP threshold (−38.9 ± 1.4 mV) were observed compared to the previous data (RMP; −56 ± 3.6 mV, AP threshold; −28 ± 1.1 mV)^22^ (Fig. 1e).

We next confirmed whether the spontaneous firing activity of serotonergic neurons depends on extrinsic synaptic inputs or intrinsic activity, we examined the contribution of major ionotropic inputs and noradrenergic α_1_ receptor^2,4^ (Fig. 1f). Bath application of glutamate and GABA_A_ receptor antagonists (20 μM 6,7-dinitroquinoxaline-2,3-(1 H, 4 H)-dione [DNQX], 50 μM DL-(-)-2-amino-5-phosphonopentanoic acid [APV], and 20 μM bicuculline) slightly decreased but did not eliminate spontaneous firing activity of serotonergic neurons (Fig. 1g,i). Similarly, α_1_ receptor antagonist (1 μM prazosin) failed to abolish the spontaneous activity (Fig. 1h,i), suggesting that serotonergic spontaneous activity shown here mainly depended on intrinsic activity of serotonergic neurons.

### L-type voltage-dependent calcium current is responsible for the spontaneous firing activity of DRN serotonergic neurons

In several types of spontaneously active neurons, such as dopaminergic neurons, the major factors for generating firing activity are T-type voltage-dependent calcium channels (VDCCs) and hyperpolarization-activated cyclic nucleotide–gated (HCN) channels^23,24^. Different from other pacemaker neurons, low-voltage activated (LVA) current and negative current injection-mediated voltage sag, which reflects the function of T-type VDCCs and HCN channels respectively, were subtle in serotonergic neurons (Supplementary Fig. S2b,c). Consistent with these observations, the blocking of T-type VDCCs (50 μM NiCl_2_) or HCN channels (20 μM ZD7288) did not decrease the serotonergic firing activity (Supplementary Fig. S2d).

**Figure 2:**
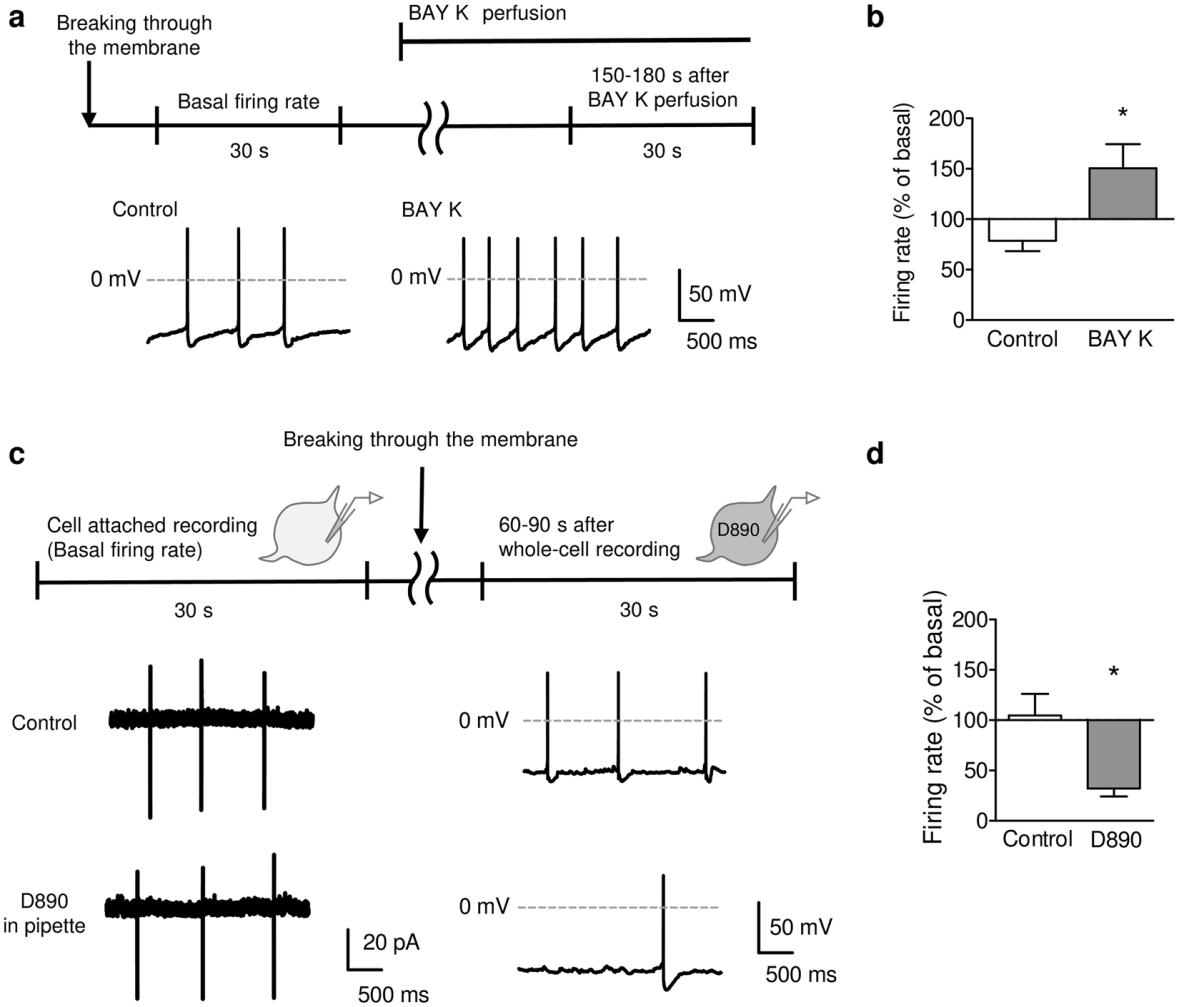
L-type voltage-dependent Ca^2+^ channel (VDCC) participates in the spontaneous activity of DRN serotonergic neurons. (**a**) Representative traces of the spontaneous firing before (left) and after (right) the application of BAY K 8644 (BAY K; 1 μM). (**b**) The effect of BAY K 8644 (BAY K; 1 μM) on the spontaneous firing rate in serotonergic neurons. The average firing rate between 150–180 s after beginning the perfusion was compared to the basal firing rate (right). *P < 0.05 *vs*. Control. (Control, *n* = 4 neurons from 2 mice; BAY K, *n* = 6 neurons from 3 mice; *P* = 0.0496, Student’s *t*-test). (**c**) To block L-type VDCC current only in the recorded neurons, D890 (0.5 mM) was added to pipette solution, and the spontaneous firing was recorded for 30 s both in cell-attached (left) and whole-cell (right) configurations. (**d**) The average firing rate between 60–90 s after beginning the whole-cell recordings was compared to that in the cell-attached recordings. *P < 0.05 *vs*. Control. (Control, *n* = 4 neurons from 3 mice; D890, *n* = 4 neurons from 3 mice; *P* = 0.0190, Student’s *t*-test). Data are presented as the mean ± S.E.M.

Recently, L-type VDCCs were recognized as machinery for generating spontaneous firing activity^25^. We next examined the involvement of L-type VDCCs in spontaneous firing activity. Blocking of L-type VDCCs with 10 μM nifedipine significantly decreased the spontaneous firing rate of serotonergic neurons (Supplementary Fig. S2a,d). On the contrary, bath application of an L-type VDCC activator, 1 μM (S)-(−)-Bay K 8644, significantly increased the spontaneous firing rate **(**Fig. 2a,b). As L-type VDCCs are widely expressed in the brain, we examined whether L-type VDCCs on serotonergic neurons or other neurons are critical for the spontaneous serotonergic activity. To test this issue, we performed intracellular application of a membrane-impermeable L-type VDCC blocker 0.5 mM D890 via a patch pipette, where L-type VDCCs are active in cell attached recordings and blocked after establishing whole-cell recordings. Intracellular application of D890 significantly decreased the spontaneous firing in whole-cell recordings compared to the basal firing rate in cell-attached recordings of the same neurons (Fig. 2c,d).

### GABA_B_ receptor-mediated signaling inhibits the L-type VDCC-mediated spontaneous firing activity

**Figure 3:**
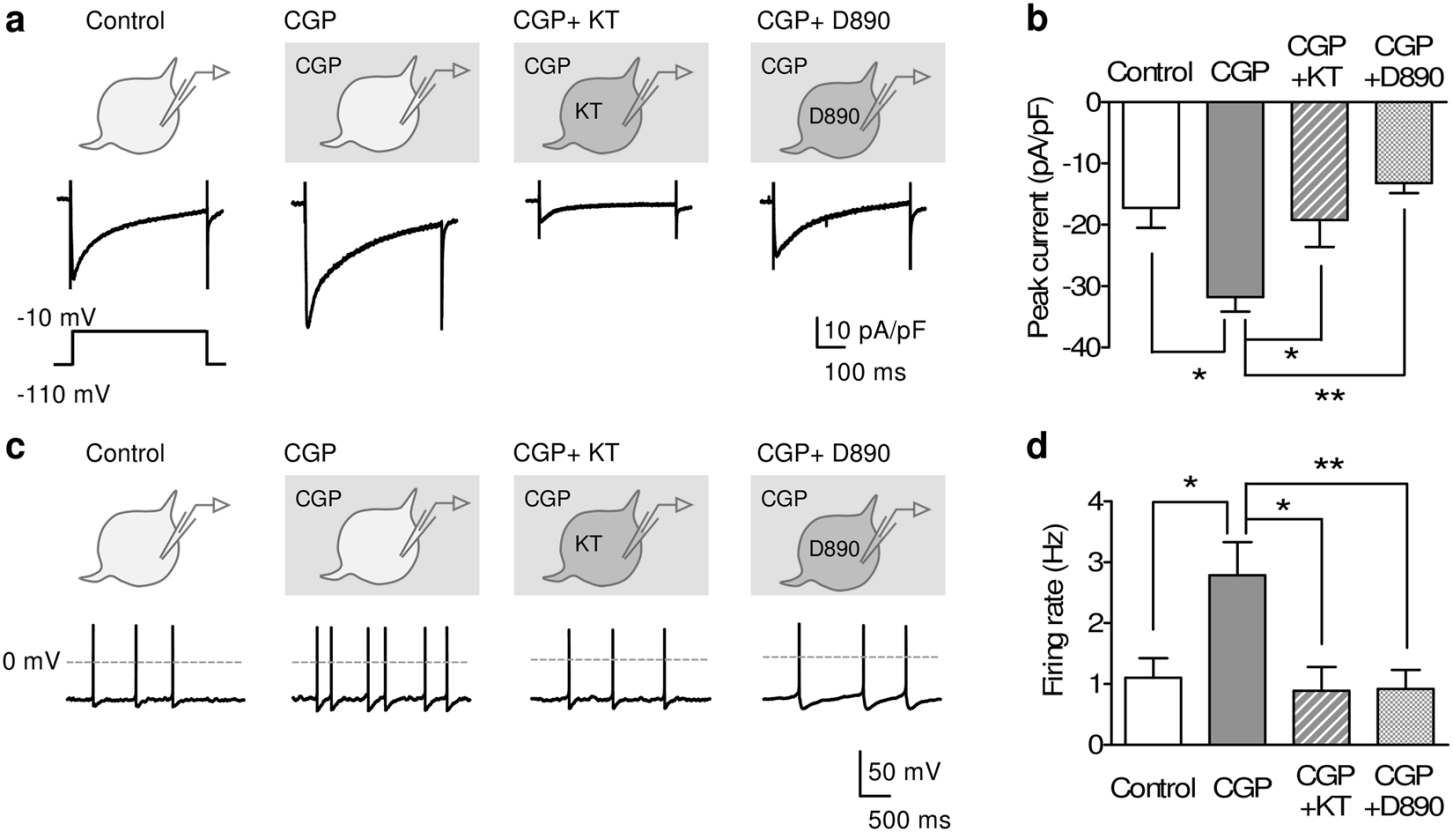
GABA_B_ receptors inhibit both L-type VDCC and pacemaker activity in DRN serotonergic neurons. (**a,b**) Representative traces (**a**) and peak current density (**b**) of high voltage activated (HVA) VDCC current in serotonergic neurons. The recordings were performed in the presence of DNQX (20 μM), APV (50 μM), bicuculline (20 μM), and tetrodotoxin (1 μM). HVA VDCC current was evoked by voltage step from −110 mV to −10 mV. CGP52432 (CGP; 10 μM) was bath-applied. KT5720 (KT; 1 μM) and D890 (0.5 mM) were applied through a patch pipette. *P < 0.05, **P < 0.01. (Control, *n* = 5 neurons from 2 mice; CGP, *n* = 8 neurons from 4 mice; CGP + KT, *n* = 8 neurons from 2 mice; CGP + D890, *n* = 7 neurons from 2 mice; one-way ANOVA; *F*(3, 24) = 6.767, *P* = 0.018; Tukey’s Multiple Comparison Test; Control vs. CGP, *P* < 0.05, CGP vs. CGP + KT, *P* < 0.05, CGP vs. CGP + D890, *P* < 0.01). (**c,d**) Representative traces (**c**) and average spontaneous firing rate (**d**) in serotonergic neurons. Recordings were performed in the presence of DNQX (20 μM), APV (50 μM), bicuculline (20 μM), WAY100635 (0.1 μM), and GR127935 (1 μM) to minimize the effects of presynaptic GABA_B_ receptor inhibition. CGP52432 (CGP; 10 μM) was bath-applied. KT5720 (KT; 1 μM) and D890 (0.5 mM) were applied through a recording pipette. *P < 0.05, **P < 0.01. (Control, *n* = 8 neurons from 4 mice; CGP, *n* = 11 neurons from 2 mice; CGP + KT, *n* = 10 neurons from 2 mice; CGP + D890, *n* = 12 neurons from 2 mice; one-way ANOVA; *F*(3, 37) = 5.112, *P* = 0.0046; Tukey’s Multiple Comparison Test; Control *vs*. CGP, *P* < 0.05, CGP *vs*. CGP + KT, *P* < 0.05, CGP *vs*. CGP + D890, *P* < 0.01). Each representative trace shows the data from different cell. Data are presented as the mean ± S.E.M.

Both in previous *in vivo* recordings and our *ex vivo* recordings in this study, the firing rate of serotonergic neurons was slower than that of other spontaneously active neurons^26,27^. Thus, we hypothesized that serotonergic firing activity receives continuous inhibition. To test this hypothesis, we examined the inhibition mechanisms of L-type VDCC current in serotonergic neurons. Besides GABA_A_ receptors, GABA_B_ receptor is a key molecule that inhibits serotonergic neurons^28^. As expected, the pharmacological blocking of GABA_B_ receptors (10 μM CGP52432) increased VDCC current (Fig. 3a,b). GABA_B_ receptors are mainly coupled with G_i/o_-type G protein and inhibit the activity of protein kinase A (PKA)^29^. GABA_B_ receptor antagonist-induced increase in VDCC current was abolished by intracellular application of a PKA inhibitor (1 μM KT5720) or an L-type VDCC blocker (0.5 mM D890) (Fig. 3a,b), suggesting that GABA_B_ receptors continuously inhibit L-type VDCC current by weakening PKA activity.

We further assessed the effects of GABA_B_ receptor-mediated inhibition of spontaneous firing activity. To exclude the involvement of ionotropic inputs and presynaptic GABA_B_ receptor-mediated change in serotonin release, we recorded the firing activity in the presence of antagonists of AMPA, NMDA, and GABA_A_ receptors and 5-hydroxytryptamine 1 (5-HT_1_) autoreceptors (20 μM DNQX, 50 μM APV, 20 μM bicuculline, 0.1 μM WAY100635, and 1 μM GR127935, termed as “antagonist mix”). Application of the antagonist mix did not affect the spontaneous firing rate of serotonergic neurons (Supplementary Fig. S3). In the presence of the antagonist mix, CGP52432 significantly increased the firing rate of serotonergic neurons. The increasing effect of CGP52432 was blocked by the intracellular application of KT5720 or D890 (Fig. 3c,d). Each drug application had no significant effect on electrophysiological characteristics, except for L-type VDCC blocker-induced elongation of AP duration^25^ (Supplementary Table S1). These results indicate that GABA_B_ receptor-mediated inhibition reduces the serotonergic spontaneous firing activity by inhibiting L-type VDCC current.

### Chronic antidepressant increases spontaneous firing activity of DRN serotonergic neurons

**Figure 4:**
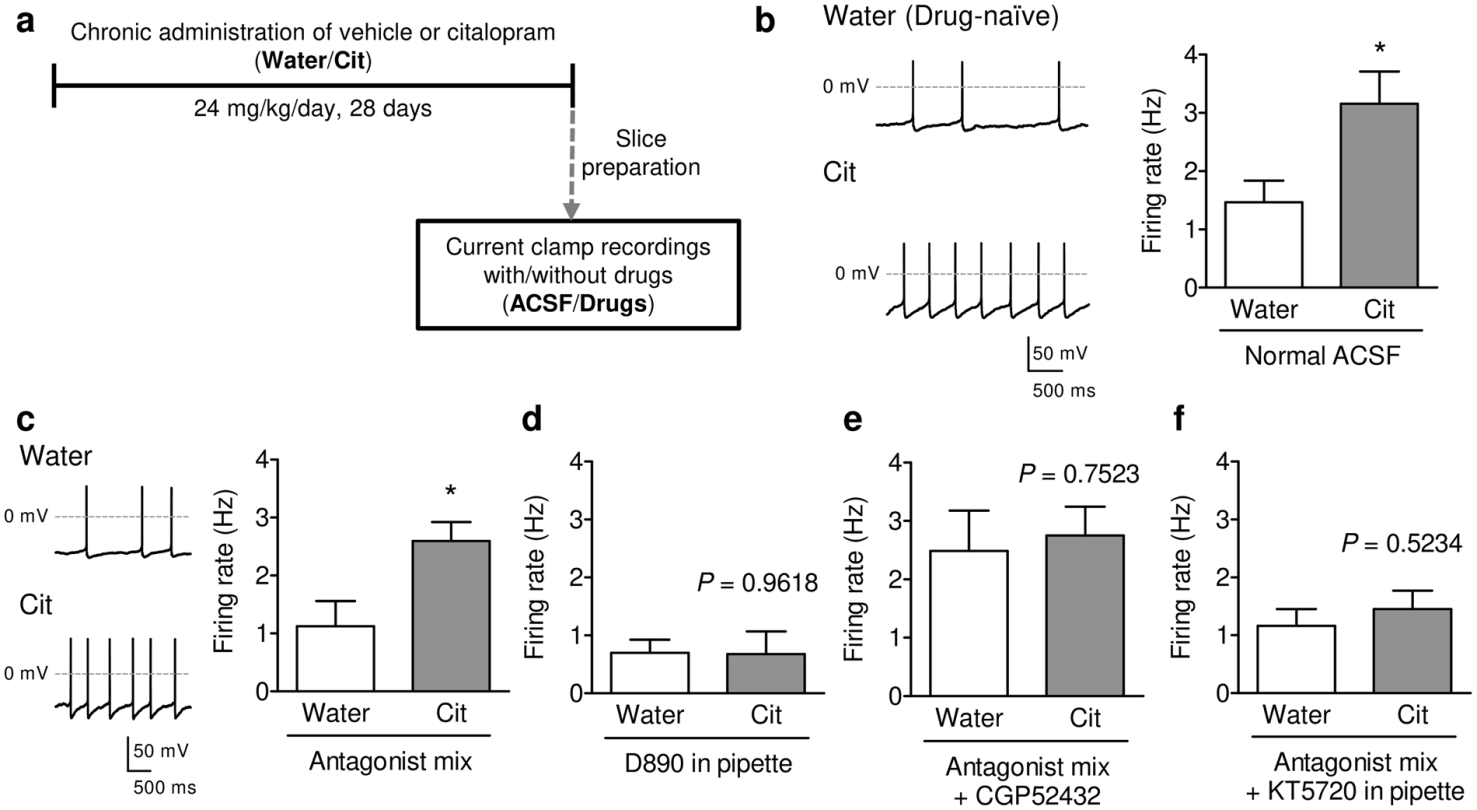
Chronic administration of citalopram increased the spontaneous firing rate of DRN serotonergic neurons. (**a**) Outline of recordings from citalopram-administrated mice. After chronic treatment with citalopram (Cit; 24 mg/kg/day) or its vehicle (Water) for 28 days, acute raphe slices were prepared, and whole-cell current clamp recordings were performed. (**b**) Representative traces (left) and average spontaneous firing rate (right) of DRN serotonergic neurons from drug-naïve (Water) and citalopram-treated (Cit) mice. *P < 0.05 *vs*. Water. (Water, *n* = 8 neurons from 4 mice; Cit, *n* = 13 neurons from 4 mice; Student’s *t*-test; *P* = 0.0491). (**c**) Representative traces (left) and average spontaneous firing rate (right) of DRN serotonergic neurons in the presence of the antagonist mix (20 μM DNQX, 50 μM APV, 20 μM Bicuculline, 0.1 μM WAY100635, and 1 μM GR127935). *P < 0.05 *vs*. Water. (Water, *n* = 8 neurons from 3 mice; Cit, *n* = 8 neurons from 4 mice; Student’s *t*-test; *P* = 0.0168). (**d**) The effects of intracellularly applied D890 on the spontaneous firing rate of DRN serotonergic neurons. *P* = 0.9618 *vs*. Water by Student’s *t*-test, Water, *n* = 13 neurons from 2 mice; Cit, *n* = 10 neurons from 2 mice. (**e**) The spontaneous firing rate of DRN serotonergic neurons in the presence of the antagonist mix and CGP52432. *P* = 0.7523 *vs*. Water by Student’s *t*-test, Water, *n* = 9 neurons from 2 mice; Cit, *n* = 12 neurons from 2 mice. (**f**) The effects of intracellularly applied KT5720 on the spontaneous firing rate of DRN serotonergic neurons in the presence of the antagonist mix. *P* = 0.5234 *vs*. Water by Student’s *t*-test, Water, *n* = 16 neurons from 2 mice; Cit, *n* = 22 neurons from 2 mice. Data are presented as the mean ± S.E.M.

It is widely accepted that potentiation of serotonergic system is important for the therapeutic activity of antidepressants^15–18^, whereas the effects and mechanisms of action of antidepressants on the activity of serotonergic neurons are unclear. We thus examined whether chronic administration of SSRIs activates DRN serotonergic activity. Mice were treated with an SSRI, citalopram (24 mg/kg/day, in drinking water) for 28 days, and *ex vivo* whole-cell recordings were performed (Fig. 4a). Under normal ACSF condition, the spontaneous activity of DRN serotonergic neurons was significantly increased by chronic treatment with citalopram compared to the drug-naïve (water-drinking) group (Fig. 4b). To further assess whether the citalopram-induced increase in the spontaneous firing rate depends on the changes in synaptic inputs, autoinhibition, or serotonergic intrinsic activity, we examined the firing activity of DRN serotonergic neurons in the presence of the antagonist mix. Even in the presence of antagonist mix, the spontaneous firing activity of serotonergic neurons in the citalopram-treated group was significantly higher than that in the drug-naïve group (Fig. 4c).

As shown in Fig. 2d, intracellular application of D890 decreased spontaneous firing activity in both the drug-naïve and citalopram-treated groups, and in this condition, chronic citalopram-induced increase in spontaneous firing rate was not observed (Fig. 4d). Similarly, in the presence of the antagonist mix, bath application of nifedipine (10 μM) also blocked chronic citalopram-induced increase in spontaneous firing activity (Supplementary Fig. S7a).

Although one of the postulated mechanisms of action of SSRI on serotonergic neurons was decreasing 5-HT_1A_ receptor-mediated autoinhibition^15,30^, SSRI also decreases GABA_B_ receptor-mediated signaling^11^. Based on this observation, we hypothesized that chronic administration of citalopram disinhibits serotonergic neurons by decreasing the GABA_B_ receptor-mediated inhibition. Consistent with the previous report^11^, GABA_B_ receptor agonist (1 mM baclofen)-induced outward current was decreased in the citalopram-treated mice, indicating decreased GABA_B_ receptor function in serotonergic neurons (Supplementary Fig. S4). As observed in Fig. 3d, blocking GABA_B_ receptors increased the spontaneous activity of serotonergic neurons in the drug-naïve mice. On the other hand, blocking of GABA_B_ receptors failed to increase firing rate in the citalopram-treated group, resulting in no difference between the drug-naïve and citalopram-treated groups (Fig. 4e). Furthermore, the intracellular application of KT5720 prevents chronic citalopram-induced increase in spontaneous activity, supporting the hypothesis that chronic citalopram activates serotonergic neurons through decreasing GABA_B_ receptor-signaling and following increase in PKA activity (Fig. 4f).

Evidence suggests that GABA_B_ receptor-mediated activation of G protein-coupled inwardly-rectifying K^+^ (GIRK) channels inhibits serotonergic neurons through hyperpolarization of the RMP^31^. However, there was no difference in pseudo RMP between the drug-naïve and citalopram-treated groups (Supplementary Table S2), indicating that chronic citalopram-induced activation of serotonergic neurons was not due to hyperpolarization of RMP. In addition, there was no positive correlation between spontaneous firing rate and pseudo RMP in “normal ACSF”, “Antagonist mix” and “Antagonist + CGP” recording conditions (Supplementary Fig. S5a,b,d). On the contrary, significant positive correlation between spontaneous firing rate and pseudo RMP was observed in “D890 in pipette” and “KT5720 in pipette” conditions, where activity of L-type VDCCs were low (Supplementary Fig. S5c,e). These results suggest that the value of RMP had little effect on spontaneous firing activity, at least when L-type VDCCs are normally active.

### Chronic antidepressant activates L-type VDCCs in DRN serotonergic neurons

**Figure 5:**
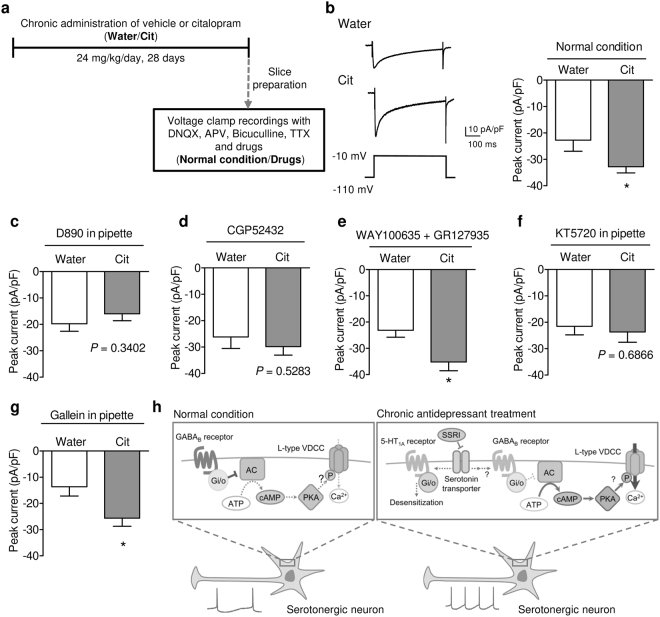
Chronic administration of citalopram increased L-type VDCC current. (**a**) Outline of recordings from citalopram-administrated mice. After chronic treatment with citalopram (Cit; 24 mg/kg/day) or its vehicle (Water) for 28 days, acute raphe slices were prepared, and whole-cell voltage clamp recordings were performed. (**b**) Representative traces (left) and peak current (right) of high voltage activated (HVA) current in DRN serotonergic neurons from drug-naïve (Water) and citalopram-treated (Cit) mice. *P < 0.05 *vs*. Water. (Water, *n* = 8 neurons from 3 mice; Cit, *n* = 9 neurons from 4 mice; Student’s *t*-test; *P* = 0.0487). (**c**) Effects of intracellularly applied D890 on HVA current in DRN serotonergic neurons. *P* = 0.3402 *vs*. Water by Student’s *t*-test, Water, *n* = 8 neurons from 2 mice; Cit, *n* = 8 neurons from 2 mice. (**d**) Effects of bath-applied CGP52432 on high voltage activated (HVA) current in DRN serotonergic neurons. *P* = 0.5283 *vs*. Water by Student’s *t*-test, Water, *n* = 14 neurons from 4 mice; Cit, *n* = 11 neurons from 2 mice. (**e**) HVA VDCC current was recorded in the presence of WAY100635 and GR127935. *P < 0.05 *vs*. Water. (Water, *n* = 11 neurons from 2 mice; Cit, *n* = 12 neurons from 2 mice; Student’s *t*-test; *P* = 0.0107). (**f**) Effects of intracellularly applied KT5720 on HVA current in DRN serotonergic neurons. *P* = 0.6866 *vs*. Water by Student’s *t*-test, Water, *n* = 10 neurons from 2 mice; Cit, *n* = 12 neurons from 2 mice. (**g**) Effects of intracellularly applied gallein (20 μM) on HVA current in DRN serotonergic neurons. *P < 0.05 *vs*. Water. (Water, *n* = 12 neurons from 2 mice; Cit, *n* = 17 neurons from 2 mice; Student’s *t*-test; *P* = 0.0172). Data are presented as the mean ± S.E.M. (**h**) In normal condition, continuous GABA_B_ receptor signaling inhibits PKA activation. Decreased PKA activity might cause inhibition of L-type VDCC activity and subsequently serotonergic firing activity. After chronic antidepressant treatment, postsynaptic GABA_B_ signaling is decreased, resulting in activation of PKA and disinhibition of L-type VDCCs. Increasing Ca^2+^ current through L-type VDCCs accelerates serotonergic firing activity.

We next examined the effects of chronic administration of citalopram in L-type VDCC current in serotonergic neurons (Fig. 5a). Voltage-clamp recordings showed that high voltage activated (HVA) current was significantly increased by chronic citalopram administration, while low-voltage activated current was not affected (Fig. 5b, Supplementary Fig. S6). The citalopram-induced increase in HVA current was abolished by the intracellular application of D890 or bath application of nifedipine (Fig. 5c, Supplementary Fig. S7b), indicating that chronic citalopram increased L-type VDCC current. Similar to spontaneous activity, blocking GABA_B_ receptors also increased VDCC current in the drug-naïve group but not in the citalopram-treated group, resulting in no difference between two groups (Fig. 5d). In the presence of nifedipine, CGP52432 did not increase HVA current in both groups, suggesting that the effect of CGP52432 in Fig. 5d was not due to the increase in other types of HVA VDCC current (Supplementary Fig. S7b). Although 5-HT_1_ autoreceptors are also G_i/o_-coupled G protein-coupled receptors, bath application of 5-HT_1_ receptor antagonists had no effect on chronic citalopram-induced increase in VDCC current (Fig. 5e).

Moreover, consistent with current clamp data, intracellular KT5720 also diminished the increasing effect of citalopram on HVA current (Fig. 5f). However, intracellular application of gallein (20 μM)-induced inhibition of G_βγ_ signaling, which is another downstream signaling by GABA_B_ receptors, did not affect the effect of chronic citalopram on VDCC current (Fig. 5g), indicating that disinhibition of PKA via decreasing GABA_B_ receptor signaling might play a critical part in SSRI-induced increase in VDCC current (Fig. 5h).

## Discussion

In this study, we determined the control mechanisms for serotonergic spontaneous firing activity. Furthermore, we found that chronic antidepressant treatment increased DRN serotonergic spontaneous activity by enhancing the spontaneous firing activity through a novel mechanism that weakens the GABA_B_ receptor-mediated inhibition of L-type VDCCs (Fig. 5h).

In the central nervous system, several types of neurons such as the midbrain dopaminergic neurons show spontaneous firing activity^26^. By using modified *ex vivo* recordings, we found that the vast majority of DRN serotonergic neurons were spontaneously active. Our data also exhibited the capability of serotonergic neurons to generate spontaneous firing activity without excitatory glutamatergic inputs or noradrenergic inputs, which are thought to be the major driving force for the firing activity of serotonergic neurons.

It is widely accepted that noradrenergic signaling on serotonergic neurons maintain serotonergic tonic activity and in *ex vivo* slices weak α_1_ receptor signaling causes low serotonergic spontaneous activity^2,32^. In accordance, effect of α_1_ antagonist on spontaneous firing activity was slight in our recording method, suggesting that spontaneous activity shown here was not due to the increase in noradrenergic inputs. On the other hand, the significant contribution of α_1_ receptor signaling on *in vivo* serotonergic activity was well established^33^. Further study will be needed to reveal the involvement of intrinsic mechanisms shown in present study on serotonergic activity control *in vivo*.

Although T-type VDCCs and HCN channels are the well-known molecular basis for generating spontaneous firing activity^24^, growing evidence suggests that L-type VDCCs, especially Cav1.3, which has a low voltage threshold^34^ (−55 mV), also contribute to maintaining the spontaneous firing activity^25^. In Cav1.3 knockout mice, the spontaneous firing activity of ventral tegmental dopaminergic neurons is lower than that in wild-type mice, whereas the activation of L-type VDCC increases the burst firing of dopaminergic neurons^35^. Consistent with these reports, the present results showed that activation of L-type VDCC also increased DRN serotonergic neuronal activity. Considering that pseudo RMPs of most of spontaneously active serotonergic neurons in this report were higher than voltage threshold of Cav1.3, present results strongly indicate the involvement of L-type VDCCs on generation of spontaneous firing activity.

For generating APs, depolarizing Ca^2+^ current through L-type VDCCs at subthreshold membrane potential plays an essential role^36^, while Ca^2+^ influx-driven subsequent signals, such as activation of Ca^2+^-activated K^+^ channels, also controls cell excitability. Ca^2+^-activated SK and BK channels are functionally coupled with L-type VDCCs and activation of these channels contributes to the generation of AHP^25,37^. SK channel-induced AHP are known to slow down firing activity and blockade of SK channels increases burst-like activity in serotonergic neurons^38^. On the contrary, BK current-induced repolarization are required for spontaneous firing activity and L-type VDCC activator-induced increase in firing rate^25,39^. While present data suggested that increasing depolarizing current through L-type VDCCs accelerate AP generation, subsequent signalings such as Ca^2+^-activated K^+^ channels-induced repolarization might be involved in the maintenance of increased tonic firing activity.

It is well-known that the local GABAergic inhibition decreases serotonergic activity^4,10^. Besides ionotropic GABA_A_ receptors, metabotropic GABA_B_ receptors are also expressed in serotonergic neurons^31^. Whereas both serotonergic and GABAergic neurons in the DRN express GABA_B_ receptors, our analysis with intracellular drug application suggest that the activation of postsynaptic GABA_B_ receptors plays an essential role in the inhibition of serotonergic neurons. While GABA_B_ receptor agonist-induced activation of GIRK channels and subsequent membrane hyperpolarization are well-studied^11,31,40^, GABA_B_ antagonist-induced depolarization was subtle in present study, suggesting that inactivation of GIRK channels had little effects on GABA_B_ antagonist-induced activation of serotonergic firing activity. However, additional researches will be required to investigate the downstream signalings of GABA_B_ receptors and how those signalings control serotonergic activity.

In present study, we showed that inhibition of postsynaptic GABA_B_ receptors in serotonergic neurons increased serotonergic activity. By contrast, previous evidences indicate that intra-DRN application of GABA_B_ agonist increases serotonin and glutamate release by stimulation of presynaptic GABA_B_ receptors^41–43^. Additionally, Milnar *et al*. have shown that co-treatment of GABA_A_ and GABA_B_ antagonists had no effect on the firing rate of phenylephrine-treated serotonergic neurons^44^. One of the reasons for this apparent discrepancy between previous reports and our results might be difference in recording condition. In the present experiments with GABA_B_ antagonist, GABAergic and glutamatergic ionotropic inputs were also blocked, where these blockers might mask changes in glutamate and GABA release through inhibition of presynaptic GABA_B_ receptors. Consequently, the effects of postsynaptic GABA_B_ receptor-antagonism might become highlighted. Further assessment of selective inhibition of GABA_B_ receptors on serotonergic neurons would be needed to elucidate GABA_B_ receptor-mediated control of serotonergic intrinsic activity.

GABA_B_ receptors interact with a variety of channels and modulate their function^45^. The interaction between GABA_B_ receptors and P/Q-type VDCCs is widely accepted as the mechanism of action for GABA_B_ receptor-mediated inhibition of neurotransmitter release^29^. Whereas, there is no consensus on whether and how GABA_B_ receptors modulate L-type VDCCs because the effects of GABA_B_ receptor activation on L-type VDCCs varies with maturation state of cells^46,47^. Evidence suggests that GABA_B_ receptors couple to G_q_ proteins and activate L-type VDCC through PKC signaling during neonatal development^48^. In this study, we used adult mice where GABA_B_ receptors might mainly couple to G_i_ proteins. Considering that PKA-mediated phosphorylation is one of the activation pathway of L-type VDCC^49^, present results indicate that GABA_B_ antagonist activates L-type VDCCs through disinhibiting PKA.

Whereas an antidepressant-induced increase in synaptic serotonin level and subsequent stimulation of postsynaptic serotonin receptors in the projection areas plays an important role in antidepressant effects^50,51^, accumulating evidence indicates that the altered DRN serotonergic activity contributes to the pathology and treatment of mental disorders^3–5^. Under stress conditions, the activity of serotonergic neurons is decreased^3,4^, and thus, chronic treatment with an antidepressant might potentiate its therapeutic effects by disinhibiting serotonergic activity.

As widely accepted, acute SSRI administration decreases activity of serotonergic neurons by increasing local serotonin levels, while continuous increase in serotonin level desensitizes 5-HT_1A_ autoreceptors and increases serotonin release^15,52^. Recent evidence indicates that dendritic serotonin release in DRN is mainly mediated by L-type VDCCs^53^, suggesting that chronic SSRI induced activation of L-type VDCC increases local serotonin release and might also facilitates disinhibition of 5-HT_1A_ autoreceptors. While chronic SSRI-induced activation of serotonergic neurons were still observed in the presence of antagonist mix, that contains a 5-HT_1A_ receptor antagonist, it is possible that chronic SSRI-induced activation of L-type VDCC further activate serotonergic neurons via disinhibiting autoreceptors. Additional research is required to identify the interaction between chronic SSRI-induced activation of L-type VDCCs and desensitization of autoreceptors.

The contribution of GABA_B_ receptors to pathogenesis and treatment for mental disorders has long been discussed. Systemic administration of GABA_B_ receptor antagonist shows serotonin-dependent antidepressant-like effect^54,55^. Furthermore, mice lacking GABA_B1b_, which preferentially exists as a postsynaptic GABA_B_ receptor, acquire stress resilience^12,56^. Consistent with a previous study^11^, our findings suggest that down regulation of postsynaptic GABA_B_ signaling in serotonergic neurons is essential for the antidepressant effect of SSRIs. On the contrary, upregulation of GABA_B_ receptor-mediated signaling produces an antidepressant-like effect in the lateral habenula and hippocampus^39,57^. These discrepancies indicate that region-specific modulation of GABA_B_ signaling is critical for therapeutic effects. In this situation, our finding of the GABA_B_ receptor-L-type VDCC signaling-mediated modulation of serotonergic function provides a novel strategy for the treatment of psychiatric disorders.

Present results suggest that chronic inhibition of serotonin transporter (SERT) decreases GABA_B_ receptor signaling in serotonergic neurons. Unlike 5-HT_1A_ receptors, GABA_B_ receptors do not internalize due to prolonged agonist exposure, and phosphorylation/dephosphorylation balance of GABA_B_ receptors determines their membrane expression and complex formation^58,59^. Evidence suggests that the chronic but not acute administration of SSRI decreases the expression of several protein kinases^60,61^. In this context, one of the possible mechanisms that explain the missing link between SERT inhibition and decrease in GABA_B_ receptor signaling is that chronic treatment with SSRI might reduce phosphorylation of GABA_B_ receptors by decreasing kinase expression. Further research will be needed to determine chronic SERT inhibition-induced signalings in serotonergic neurons.

In conclusion, the current *ex vivo* electrophysiological investigations indicated that DRN serotonergic neurons possess spontaneous firing activity, in which L-type VDCC is a key modulator. This spontaneous activity received tonic inhibition through GABA_B_ receptor-mediated inhibition of L-type VDCCs, and chronic administration of SSRI weakened this inhibition. Our findings provide a new mechanism for the regulation of serotonergic activity and raise the possibility that postsynaptic GABA_B_ receptor-mediated inhibition of L-type VDCCs in serotonergic neurons might be a promising target for the treatment of psychiatric disorders.

## Methods

### Reagents

DL-2-Amino-5-phosphonopentanoic acid (DL-APV; a selective NMDA antagonist; Sigma-Aldrich, St-Louis, MO, USA), WAY100635 (a 5-HT_1A_ antagonist; Abcam Biochemicals, Cambridge, UK), GR127935 (a selective 5-HT_1B_ antagonist; Abcam Biochemicals), CGP52432 (a selective GABA_B_ antagonist; Abcam Biochemicals), and tetrodotoxin (a selective voltage-dependent Na^+^ channel blocker; Sigma-Aldrich) were dissolved in water. 6,7-dinitroquinoxaline-2,3(1 H,4 H)-dione (DNQX; an AMPA (non-NMDA) antagonist; Tocris Bioscience, Bristol, UK), bicuculline (a selective GABA_A_ antagonist; Enzo Life Science, Farmingdale, NY, USA), prazosin (an α_1_ receptor antagonist; Sigma-Aldrich), ZD7288 (a selective hyperpolarization-activated cyclic nucleotide–gated channel blocker; Cayman Chemical Company, Ann Arbor, MI, USA), (S)-(-)-Bay K 8644 (Bay K 8644; an L-type voltage-dependent Ca^2+^ channel (VDCC) activator; Santa Cruz Biotechnology, Santa Cruz, CA, USA), nifedipine (an L-type VDCC blocker; Wako Pure Chemical Industries, Osaka, Japan), KT5720 (a selective PKA inhibitor; Wako Pure Chemical Industries), and gallein (a selective G_βγ_ inhibitor; Sigma-Aldrich) were dissolved in dimethyl sulfoxide (DMSO). Baclofen (a selective GABA_B_ agonist; Wako Pure Chemical Industries) was directly dissolved in artificial cerebrospinal fluid (ACSF). D890 (a quaternary derivative of methoxyverapamil acts as a membrane-impermeable L-type VDCC blocker; Abcam Biochemicals) was directly dissolved in the pipette solution. Stock solutions were stored at −20 °C until use and dissolved in ACSF or pipette solution for recording. The final concentration of DMSO in ACSF and pipette solution was lower than 0.05%.

### Animals

All animal care and experimental procedures were conducted in accordance with the ethical guidelines of the Kyoto University Animal Research Committee and were approved by the Animal Research Committee, Graduate School of Pharmaceutical Sciences, Kyoto University (Approval number: 13–41). Male C57BL/6 J mice were purchased from Nihon SLC (Shizuoka, Japan) and singly housed at a constant ambient temperature of 24 ± 1 °C on a 12-h light-dark cycle with access to food and water *ad libitum*.

For chronic antidepressant treatment, citalopram hydrobromide (FWD Chemicals, Shanghai, China) was dissolved in drinking water (0.2 mg/mL) and administrated for 28 days. Water consumption was approximately 3–4 mL/day/mouse, resulting in average dose at 24 mg/kg/day. The drug containing drinking water was shielded from light and changed every 3–5 day.

### Preparation of acute raphe slices for electrophysiological analysis

Male 11–12-week-old mice were deeply anesthetized with isoflurane and decapitated. The brains were rapidly collected in ice-cold cutting solution (composition in mM; 120 NMDG-Cl, 2.5 KCl, 26 NaHCO_3_, 1.25 NaH_2_PO_4_, 0.5 CaCl_2_, 7 MgCl_2_, 15 D-glucose, and 1.3 ascorbic acid, pH 7.2). Coronal midbrain slices (200-μm thick) were prepared with a vibratome (VT1000S, Leica, Wetzlar, Germany). Knife speed and frequency were 0.025–0.05 mm/s and 60–70 Hz, respectively. Slices were recovered in oxygenated ACSF (composition in mM; 124 NaCl, 3 KCl, 26 NaHCO_3_, 1 NaH_2_PO_4_, 2.4 CaCl_2_, 1.2 MgCl_2_, and 10 D-glucose, pH 7.3) at 32 °C for at least 1 h before recording. After recovery, individual slices were transferred to a recording chamber with continuous perfusion of oxygenated ACSF at a flow rate of 1–2 mL/min. ACSF were warmed to keep the recording chamber at 27 ± 1 °C. Recordings were performed only within 4 hours after recovery.

### Electrophysiological recordings

Electrophysiological recordings were performed as previously described^10^ with several modifications. Electrophysiological recordings were performed with an EPC9 amplifier (HEKA, Pfalz, Germany), and the data were recorded using Patchmaster software (HEKA). The resistance of the electrodes was 3–6 MΩ when filled with the internal solution (composition in mM; 140 K-gluconate, 5 KCl, 10 HEPES, 2 Na-ATP, 2 MgCl_2_, and 0.2 EGTA, pH 7.3 adjusted with KOH for current clamp recordings, and 120 CsMeSO_4_, 15 CsCl, 8 NaCl, 10 HEPES, 2 Na-ATP, 0.3 Na-GTP, 0.2 EGTA, 10 TEA-Cl, and 5 QX-314, pH 7.3 adjusted with CsOH for voltage clamp recordings). Individual neurons were visualized with a microscope equipped with a 40 × water-immersion objective lens (Carl Zeiss, Jena, Germany) and a CCD camera. The series resistance was compensated by 70% and maintained within 20 MΩ.

The spontaneous firing was examined in cell-attached or whole-cell current-clamp recordings. Cell-attached recordings were performed at a holding potential of 0 mV. In whole-cell current-clamp recordings, the current was held at 0 pA. For comparing the spontaneous activity between different neurons, spontaneous firing activity was recorded for 30 s after stabilization. To examine the change in spontaneous firing within a neuron, the average firing rate in the first 30 s was considered as the basal firing rate. VDCC current was recorded under a voltage-clamp condition in the presence of DNQX (20 μM), APV (50 μM), bicuculline (20 μM), and tetrodotoxin (1 μM), and was generated by depolarizing voltage steps from −110 mV to −40 mV or −10 mV. Membrane potential between recordings was held at −70 mV. Baclofen-induced current was recorded in the presence of DNQX (20 μM), APV (50 μM), and bicuculline (20 μM), and the holding potential was set at −50 mV.

### Single-cell reverse transcription-polymerase chain reaction (RT-PCR)

Single-cell RT-PCR was performed as previously described^10^. After the whole-cell recording, the contents of the cell were aspirated into the recording pipette and harvested in a sampling tube. The collected samples were reverse-transcribed using a ReverTra Ace RT kit (TOYOBO, Tokyo, Japan) and amplified with Blend Taq (TOYOBO, Tokyo, Japan). The oligonucleotide primers used were 5′-TAGGCTTAGCGTCTCTGGGA-3′ and 5′-AAGGCCGAACTCGATTGTGA-3′ for *Tph2*; 5′-GGCCTGAAGATCTGTGGCTT-3′ and 5′-CAGAACCTTGGTGGAGCGAT-3′) for *Gad1*; 5′-ATGCAGAGCTGCAACCAGAT-3′ and 5′-GCCTCAAACCCAGTAGTCCC-3′ for *Gad2*; 5′-CCGCTGATCCTTCCCGATAC-3′ and 5′-CGACGTTGGCTGTGAACTTG-3′ for *Eno2* as a neuronal marker. PCR products were analyzed using agarose gel electrophoresis. Only when *Tph2* mRNA expression was detected, the data was used for analysis (Fig. 1a).

### Statistics

All data are presented as the mean ± standard error of mean (S.E.M). Statistical analysis was performed by GraphPad Prism 5 (GraphPad, San Diego, CA, USA). Differences with *P* < 0.05 were considered significant. The differences between two groups were compared by two-tailed Student’s *t*-test. When comparing differences within the cell, two-tailed paired *t*-test was used for analysis. The differences between more than three groups were compared by one-way analysis of variance (ANOVA) with *post hoc* Tukey’s Multiple Comparison Test. When examining the time-course, two-way ANOVA for repeated measures was used for analysis. For correlation analysis, Pearson correlation coefficients were used.

### Data availability

All data generated or analysed during this study are included in this published article and its Supplementary Figure files.

## Electronic supplementary material

Supplementary Figures and Tables
