# Supplementary material for: Chronic antidepressant potentiates spontaneous activity of dorsal raphe serotonergic neurons by decreasing GABAB receptor-mediated inhibition of L-type calcium channels
Source: Sci Rep. 2017 Oct 19;7:13609. doi: 10.1038/s41598-017-13599-3 (PMC5648823; doi:10.1038/s41598-017-13599-3)
Supplement: Supplementary file 1 — Supplementary Figures and Tables [file 41598_2017_13599_MOESM1_ESM.pdf]

Chronic antidepressant potentiates spontaneous activity of dorsal raphe serotonergic neurons by decreasing $GABA_B$ receptor-mediated inhibition of L-type calcium channels

Nozomi Asaoka[1], Naoya Nishitani[1], Haruko Kinoshita[1], Hiroyuki Kawai[1], Norihiro Shibui[1], Kazuki Nagayasu[1*], Hisashi Shirakawa[1], Takayuki Nakagawa[2] & Shuji Kaneko[1*]

Supplementary Figures and Tables

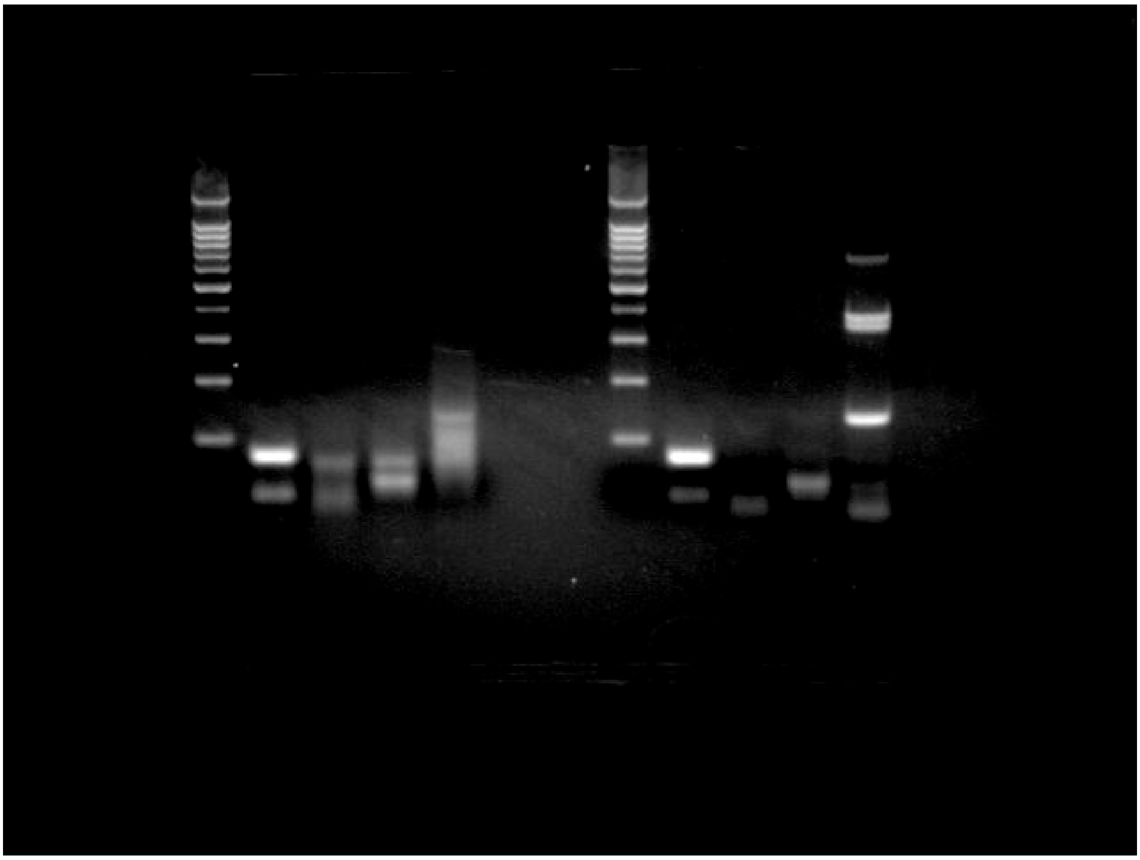

Supplementary Fig. S1. Uncropped image of agarose gel electrophoresis shown in Fig. 1a (Right side of the image). PCR Samples (amplicon size in bp): Size marker, Tph2 (83), Gad1 (288), Gad2 (164), Eno2 (144) (from left to right).

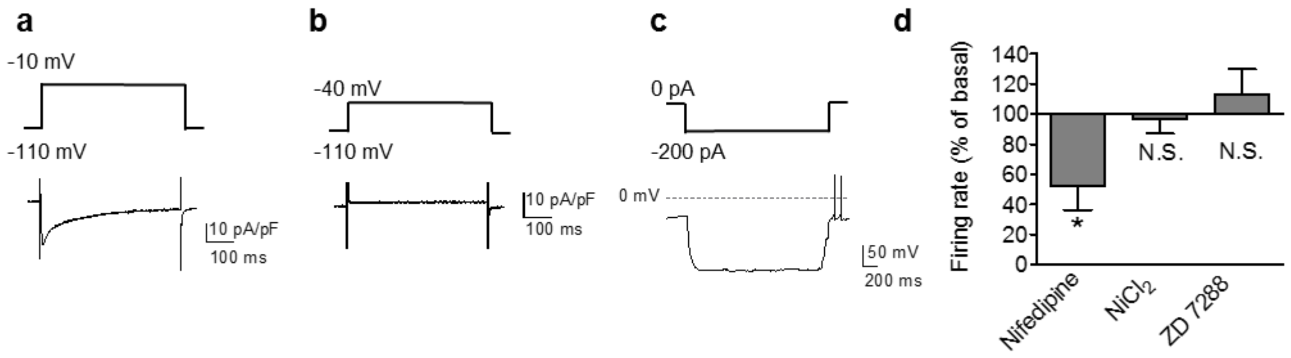

Supplementary Fig. S2. L-type voltage dependent voltage-dependent Ca^{2+} channel (VDCCs) but not T-type VDCCs and hyperpolarization-activated cyclic nucleotide-gated (HCN) channels modulate serotonergic spontaneous firing activity. (**a**) Representative trace of high voltage activated (HVA) current evoked by a voltage step from -110 mV to -10 mV. (**b**) Representative trace of low voltage activated (LVA) current evoked by a voltage step from -110 mV to -40 mV. (**c**) Representative trace of the membrane potential change by negative current injection (-200 pA). (**d**) The changes in the spontaneous firing rate before and after the application of nifedipine. *$P < 0.05$. (10 μM, n = 5 neurons from 3 mice, P = 0.0363 by paired t-test), $NiCl_2$ (50 μM, n = 6 neurons from 2 mice, P = 0.7558 by paired t-test), or ZD7288 (20 μM, n = 3 neurons from 2 mice, P = 0.5385 by paired t-test). Data are presented as the mean ± S.E.M.

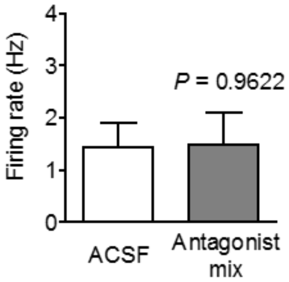

Supplementary Fig. S3. Bath application of “antagonist mix” did not affect serotonergic spontaneous firing activity. Serotonergic spontaneous firing activity was recorded in normal ACSF condition or in the presence of antagonist mix (20 μM DNQX, 50 μM APV, 20 μM bicuculline, 0.1 μM WAY100635, and 1 μM GR127935). (Water, n = 13 neurons from 5 mice; Cit, n = 9 neurons from 3 mice, P = 0.9622 *vs.* Water by Student’s t-test,.) Data are presented as the mean ± S.E.M.

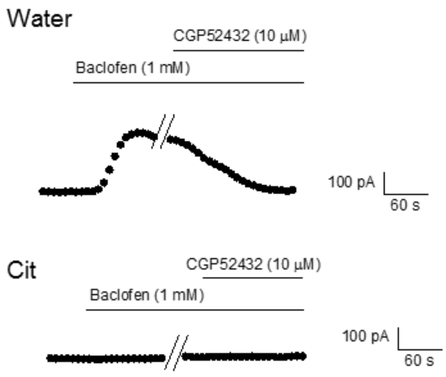

Supplementary Fig. S4. Chronic administration of citalopram decreased postsynaptic $GABA_B$ receptor signaling in serotonergic neurons. Representative traces of baclofen (1 mM)-induced current in serotonergic neurons from drug-naïve (Water) and citalopram-treated (Cit) mice. The holding voltage was set at -50 mV. *$P < 0.05$ *vs.* Water. (Similar responses were observed at least 4 times).

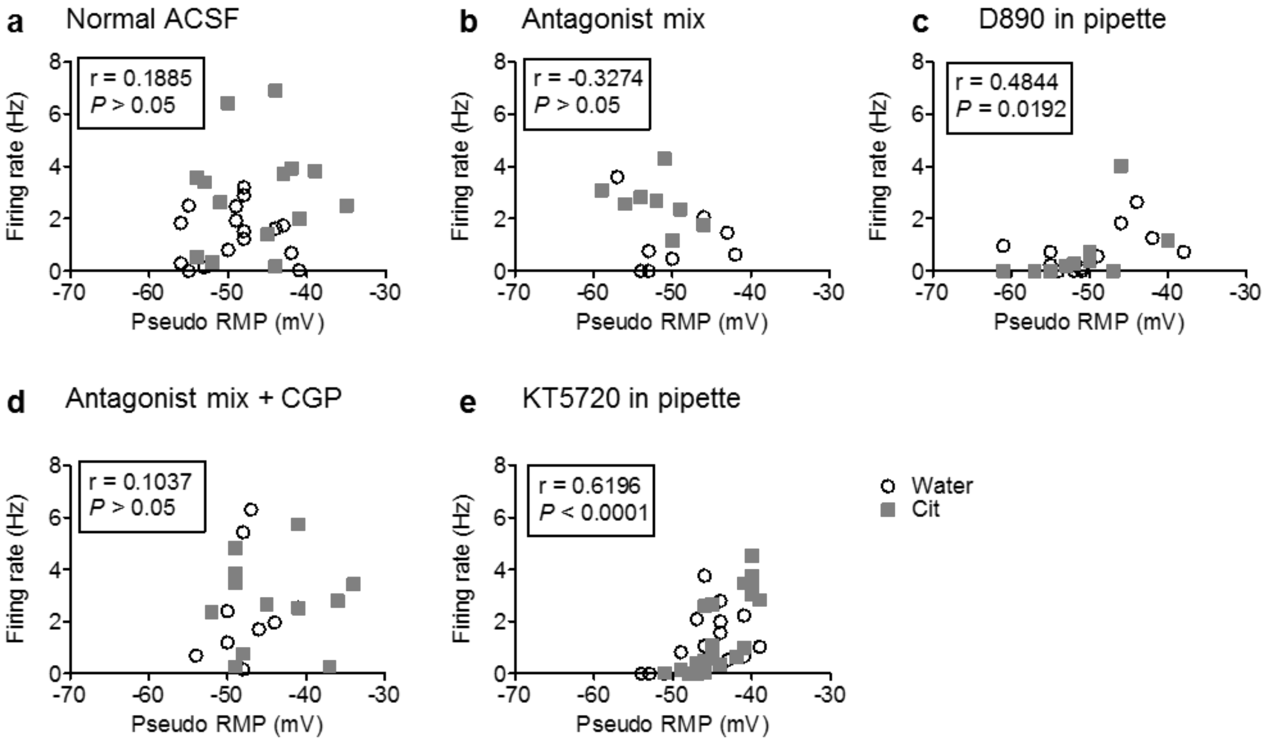

Supplementary Fig. S5. Relationship between spontaneous firing rate and pseudo RMP of serotonergic neurons from drug-naïve (Water) and citalopram-treated (Cit) mice. Spontaneous firing rate was plotted versus pseudo RMP and Pearson correlation analysis were used to determine the relationship.

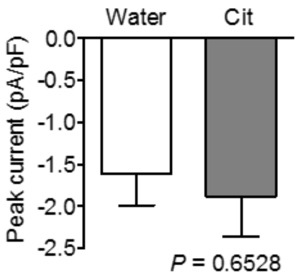

Supplementary Fig. S6. Chronic administration of citalopram did not affect low voltage activated (LVA) VDCC current. LVA VDCC current in serotonergic neurons was recorded by a voltage step from -110 mV to -40 mV. (Water, n = 9 neurons from 3 mice; Cit, n = 9 neurons from 4 mice, P = 0.6528 *vs.* Water by Student's *t*-test.) Data are presented as the mean ± S.E.M.

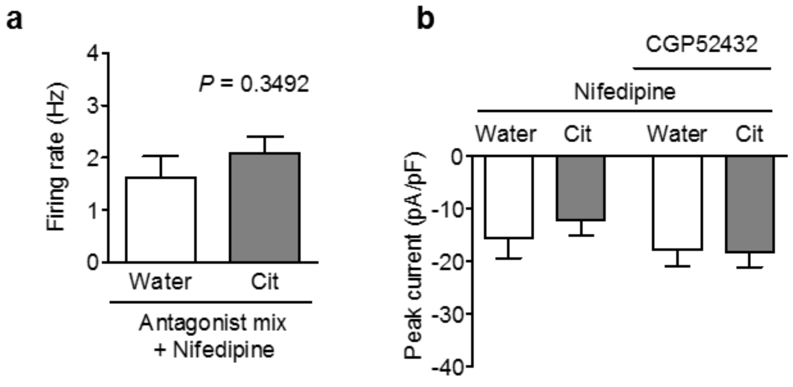

Supplementary Fig. S7. Nifedipine blocked the increasing effects of citalopram on spontaneous firing activity and VDCC current. (**a**) Effects of nifedipine on the spontaneous firing rate of DRN serotonergic neurons. (Water, n = 18 neurons from 4 mice; Cit, n = 25 neurons from 5 mice, P = 0.3492 *vs.* Water by Student's t-test.) (**b**) Effects of nifedipine and CGP52432 on HVA VDCC current in DRN serotonergic neurons from drug-naïve (Water) and citalopram-treated (Cit) mice. (one-way ANOVA; $F_{3, 34}$ = 0.7042, P = 0.5562; Tukey's Multiple Comparison Test; P > 0.05 (all pairs); Water (Nifedipine), n = 7 neurons from 3 mice; Cit (Nifedipine), n = 8 neurons from 3 mice; Water (Nifedipine + CGP), n = 13 neurons from 2 mice; Cit (Nifedipine + CGP), n = 10 neurons from 2 mice). Data are presented as the mean ± S.E.M.

	Spontaneous firing frequency (Hz)	Pseudo RMP (mV)	AP threshold (mV)	AP amplitude (mV)	AP duration (ms)	AHP amplitude (mV)
Control	1.1 ± 0.3 (*n* = 8)	-47.9 ± 1.6 (*n* = 8)	-38.0 ± 1.7 (*n* = 7)	86.2 ± 2.6 (*n* = 7)	3.1 ± 0.3 (*n* = 7)	-20.8 ± 1.4 (*n* = 7)
CGP52432	2.8 ± 0.5[1] (*n* = 11)	-43.3 ± 1.2 (*n* = 11)	-36.0 ± 0.7 (*n* = 10)	78.7 ± 1.6 (*n* = 10)	2.6 ± 0.1 (*n* = 10)	-18.4 ± 0.7 (*n* = 10)
CGP52432 + KT5720	0.9 ± 0.4[2] (*n* = 10)	-46.2 ± 1.3 (*n* = 10)	-37.7 ± 1.3 (*n* = 7)	77.7 ± 7.0 (*n* = 7)	3.0 ± 0.3 (*n* = 7)	-17.6 ± 0.7 (*n* = 7)
CGP52432 + D890	0.9 ± 0.3[2] (*n* = 12)	-46.2 ± 0.7 (*n* = 12)	-35.4 ± 1.1 (*n* = 10)	74.0 ± 2.8 (*n* = 10)	5.2 ± 0.4[1, 2, 3] (*n* = 10)	-19.0 ± 0.8 (*n* = 10)

1; $P < 0.05$ compared to “control” group. 2; $P < 0.05$ compared to “CGP52432” group.
3; $P < 0.05$ compared to “CGP52432 + KT5720” group.
The differences were compared by one-way analysis of variance (ANOVA) with post hoc Tukey's Multiple Comparison Test.

Supplementary Table S1 Electrophysiological characters of serotonergic neurons related to Figure 3.

		Spontaneous firing frequency (Hz)	Pseudo RMP (mV)	AP threshold (mV)	AP amplitude (mV)	AP duration (ms)	AHP amplitude (mV)
ACSF	Water	1.5 ± 0.4 (*n* = 8)	-47.7 ± 1.4 (*n* = 8)	-36.4 ± 1.3 (*n* = 7)	85.6 ± 1.9 (*n* = 7)	2.7 ± 0.2 (*n* = 7)	-23.0 ± 1.3 (*n* = 7)
	Cit	3.1 ± 0.5[1] (*n* = 13)	-45.8 ± 1.7 (*n* = 13)	-34.8 ± 1.3 (*n* = 13)	80.4 ± 1.7 (*n* = 13)	2.8 ± 0.1 (*n* = 13)	-23.6 ± 1.0 (*n* = 13)
	P value	*P* = 0.0394	*P* > 0.05	*P* > 0.05	*P* > 0.05	*P* > 0.05	*P* > 0.05
Antagonist mix	Water	1.1 ± 0.4 (*n* = 8)	-49.7 ± 1.9 (*n* = 8)	-38.3 ± 2.2 (*n* = 6)	83.8 ± 2.4 (*n* = 6)	2.7 ± 0.1 (*n* = 6)	-22.7 ± 1.0 (*n* = 6)
	Cit	2.6 ± 0.3[1] (*n* = 8)	-52.1 ± 1.4 (*n* = 8)	-42.2 ± 2.1 (*n* = 8)	87.3 ± 2.0 (*n* = 8)	2.5 ± 0.1 (*n* = 8)	-22.0 ± 1.0 (*n* = 8)
	P value	*P* = 0.0168	*P* > 0.05	*P* > 0.05	*P* > 0.05	*P* > 0.05	*P* > 0.05
D890 in pipette	Water	0.7 ± 0.2 (*n* = 13)	-50.7 ± 1.9 (*n* = 13)	-40.9 ± 2.4 (*n* = 9)	76.3 ± 4.0 (*n* = 9)	4.7 ± 0.4 (*n* = 9)	-20.3 ± 2.0 (*n* = 9)
	Cit	0.7 ± 0.4 (*n* = 10)	-51.1 ± 1.9 (*n* = 10)	-39.1 ± 2.2 (*n* = 6)	68.0 ± 3.0 (*n* = 6)	4.1 ± 0.6 (*n* = 6)	-16.0 ± 2.0 (*n* = 6)
	P value	*P* > 0.05	*P* > 0.05	*P* > 0.05	*P* > 0.05	*P* > 0.05	*P* > 0.05
Antagonist mix + CGP52432	Water	2.5 ± 0.7 (*n* = 9)	-47.5 ± 1.2 (*n* = 9)	-40.9 ± 1.2 (*n* = 9)	80.3 ± 1.1 (*n* = 9)	3.0 ± 0.3 (*n* = 9)	-18.1 ± 1.3 (*n* = 9)
	Cit	2.7 ± 0.5 (*n* = 12)	-44.2 ± 1.8 (*n* = 12)	-36.2 ± 1.9 (*n* = 12)	74.5 ± 2.9 (*n* = 12)	3.0 ± 0.2 (*n* = 12)	-16.1 ± 0.5 (*n* = 12)
	P value	*P* > 0.05	*P* > 0.05	*P* > 0.05	*P* > 0.05	*P* > 0.05	*P* > 0.05
KT5720 in pipette	Water	1.2 ± 0.3 (*n* = 16)	-46.6 ± 1.2 (*n* = 16)	-38.5 ± 1.0 (*n* = 11)	83.5 ± 2.9 (*n* = 11)	2.7 ± 0.2 (*n* = 11)	-17.9 ± 1.0 (*n* = 11)
	Cit	1.4 ± 0.3 (*n* = 22)	-44.1 ± 0.7 (*n* = 22)	-35.7 ± 0.6[1] (*n* = 20)	79.9 ± 2.3 (*n* = 20)	2.7 ± 0.1 (*n* = 20)	-16.9 ± 0.5 (*n* = 20)
	P value	*P* > 0.05	*P* > 0.05	*P* = 0.0161	*P* > 0.05	*P* > 0.05	*P* > 0.05

1; $P < 0.05$ compared to "Water (drug-naïve)" group.
The differences were compared by paired t-test.

Supplementary Table S2 Electrophysiological characters of serotonergic neurons from drug-naïve or citalopram-treated mice.
